# The Ethnic-Specific Spectrum of Germline Nucleotide Variants in DNA Damage Response and Repair Genes in Hereditary Breast and Ovarian Cancer Patients of Tatar Descent

**DOI:** 10.3389/fonc.2018.00421

**Published:** 2018-10-02

**Authors:** Olga I. Brovkina, Leila Shigapova, Daria A. Chudakova, Marat G. Gordiev, Rafael F. Enikeev, Maxim O. Druzhkov, Dmitriy S. Khodyrev, Elena I. Shagimardanova, Alexey G. Nikitin, Oleg A. Gusev

**Affiliations:** ^1^Federal Research and Clinical Center, Federal Medical-Biological Agency of Russia, Moscow, Russia; ^2^Kazan (Volga Region) Federal University, Kazan, Russia; ^3^Institute of Natural and Mathematical Sciences, Massey University, Auckland, New Zealand; ^4^Republican Clinical Oncology Dispensary of the Ministry of Health of the Republic of Tatarstan, Kazan, Russia; ^5^Pulmonology Research Institute, Federal Medical-Biological Agency of Russia, Moscow, Russia; ^6^RIKEN, Yokohama, Japan

**Keywords:** breast cancer, *BRCA1*, *BRCA2*, *CDK12*, homologous recombination repair, next-generation sequencing, ovarian cancer

## Abstract

The Russian population consists of more than 100 ethnic groups, presenting a unique opportunity for the identification of hereditary pathogenic mutations. To gain insight into the landscape of heredity pathogenic variants, we employed targeted next-generation sequencing to analyze the germline mutation load in the DNA damage response and repair genes of hereditary breast and ovary cancer syndrome (HBOCS) patients of Tatar ethnicity, which represents ~4% of the total Russian population. Several pathogenic mutations were identified in DNA double-strand break repair genes, and the spectrum of these markers in Tatar patients varied from that previously reported for patients of Slavic ancestry. The CDK12 gene encodes cyclin-dependent kinase 12, the key transcriptional regulator of the genes involved in DNA damage response and repair. C*DK12* analysis in a cohort of HBOCS patients of Tatar decent identified a c.1047-2A>G nucleotide variant in the C*DK12* gene in 8 of the 106 cases (7.6%). The c.1047-2A>G nucleotide variant was identified in 1 of the 93 (1.1%) HBOCS patients with mixed or unknown ethnicity and in 1 of the 238 (0.42%) healthy control patients of mixed ethnicity (Tatars and non-Tatars) (*p* = 0.0066, OR = 11.18, CI 95% = 1.53–492.95, Tatar and non-Tatar patients vs. healthy controls). In a group of mixed ethnicity patients from Tatarstan, with sporadic breast and/or ovarian cancer, this nucleotide variant was detected in 2 out of 93 (2.2%) cases. In a cohort of participants of Slavic descent from Moscow, comprising of 95 HBOCS patients, 80 patients with sporadic breast and/or ovarian cancer, and 372 healthy controls, this nucleotide variant was absent. Our study demonstrates a strong predisposition for the C*DK12* c.1047-2A>G nucleotide variant in HBOCS in patients of Tatar ethnicity and identifies C*DK12* as a novel gene involved in HBOCS susceptibility.

## Introduction

Ovarian (OC) and breast (BC) cancers are the leading causes of oncological mortality in women worldwide (1). Both cancers are highly heterogeneous with a strong hereditary component, as ~10–15% of OC and 5–7% of BC cases are hereditary (2). The hereditary predisposition for these cancers (hereditary breast and ovarian cancer syndrome, HBOCS) is caused by germline mutations in several genes, primarily those linked to DNA damage recognition and repair. Early diagnosis reduces the disease-associated mortality rate. Therefore, genetic testing for HBOCS predisposition would be a beneficial addition to routine clinical practices.

Currently, genetic risk assessment for HBOCS profiles pathogenic DNA nucleotide variants for a panel of candidate genes. This approach allows for a stratification of patients into different subgroups with tailored therapies and for the identification of individuals at risk of HBOCS before there is a clinical manifestation of the disease (3). Importantly, the distribution pattern of the pathogenic DNA nucleotide variants may differ significantly across different ethnic populations due to the “founder effect” (4), and genetic tests developed for European populations may be clinically uninformative for patients of non-European ancestry. Therefore, genetic testing of patients with diverse ethnic backgrounds should be performed using a panel of markers established specifically for their ethnic group. In Russia, most genetic risk assessment tests for HBOCS include a panel of pathogenic nucleotide variants that are common among patients of European descent such as 5382insC, C61G, 185delAG, 4154delA, and 2080delA variants in the *BRCA1* gene. While those nucleotide variants have been comprehensively characterized in Russian Slavic populations (2, 5–7), recent data indicates that many of them are absent in patients from the Tatar ethnic origin (8). Therefore, there is a clear clinical demand for identification of novel HBOCS predisposing nucleotide variants specific for the Tatar population.

Genomic instability is a hallmark of cancer (9). Defects in DNA damage recognition and repair are associated with a plethora of malignancies including prostate cancer, ovarian cancer, leukemia, and breast cancer (10–13). In hereditary cancers, a major cause of genomic instability is the inability of the cell to repair DNA damage properly due to germline mutations in genes encoding DNA-repair proteins.

In mammals, the major pathways for DNA repair are base-excision repair (BER), nucleotide-excision repair (NER), non-homologous end joining (NHEJ), and homologous recombination repair (HRR) (14). DNA double-strand breaks (DSBs) are repaired by NHEJ and HRR. The NHEJ pathway orchestrates re-ligation of DSB ends, after removal of damaged nucleotides (15). The HRR pathway repairs DSBs using undamaged homologous DNA as a template sequence. NHEJ is less accurate than HRR, while HRR is characterized by high fidelity and is, therefore, essential for the maintenance of genomic integrity. For many of the genes involved in the HRR pathway, an association with tumorigenesis was clearly demonstrated in both sporadic and hereditary cancers.

The role of DSB repair pathway genes in susceptibility to breast and ovarian cancer has been heavily investigated. The panel of the genes contributing to HBOCS includes several DSB repair genes such as *BRCA1, BRCA2*, and others (16–19). Mutations in *BRCA1* and *BRCA2* genes, which inactivate the corresponding proteins and compromise the function of HRR pathways, contribute to ~20–25% of HBOCS cases (20, 21). However, the remaining cases are comprised of patients with functional BRCA1 and BRCA2 proteins (BRCA1/2 negative HBOCS). For many of these cases, none of the currently used diagnostic markers are present and the predisposition genes remain obscure.

A number of publications indicate that Cyclin-dependent kinase 12 (CDK12), also known as KIAA0904, CRK7, CRKR, or CRKRS, is involved in human tumorigenesis (22). There are recurrent somatic mutations in the *CDK12* gene identified in OC (23). Moreover, somatic mutations resulting in *CDK12* inactivation are associated with genomic instability in OC (24). *CDK12* is also an emerging candidate BC tumor suppressor gene (25).

CDK12 is a serine/threonine protein kinase, a member of the cyclin-dependent kinase family. It is a multifunctional protein involved in many cellular processes such as alternative last exon mRNA splicing (21), embryonic stem cells renewal (26), cellular stress-response (27), and regulation of global transcription by targeting of RNA polymerase II, the polymerase that transcribes mRNA for protein-coding genes (28). Importantly, CDK12 is a key regulator of expression of DNA damage response genes. While depletion of CDK12 does not significantly affect global transcription, it dramatically diminishes transcription of the genes involved in DNA damage response and repair pathways including *BRCA1*, a gene established to convey HBOCS predisposition. Furthermore, cells with CDK12 depletion are more sensitive to DNA damaging agents and exhibit a higher rate of spontaneous DNA damage (29). Thus, CDK12 plays a pivotal role in the maintenance of genomic stability (30). However, currently there is little data on the role of *CDK12* germline mutations in HBOCS pathogenesis. We propose that *CDK12* is a candidate gene for HBOCS predisposition.

The aim of this study was to identify a panel of DNA nucleotide variant markers for HBOCS syndrome genetic screening in patients of Tatar ethnic origin. Using Targeted Next Generation Sequencing, we tested a panel of markers in the ATM, BARD1, BRCA1, BRCA2, CDH1, CDK4, CDK12, CDKN2A, CFTR, CHEK1, CHEK2, CTNNA1, EPCAM, FANCI, FANCJ/BRIP1, FANCL, MLH1, MSH2, MSH6, MUTYH, PALB2, PARP1, PDGFRA, PMS2, PPP2R2A, PRSS1, RAD51B, RAD51C, RAD51D, RAD54L, SPINK1, STK11, TP53, and XRCC3 genes of 199 HBOCS patients (Tatars and non-Tatars from the Volga District, Tatarstan Republic). Several pathogenic nucleotide variant markers were identified in the BRCA1, BRCA2, CDH1, CDK12, CHEK2, FANCI, MUTYH, MSH2, and RAD51C genes. The marker distribution profile in Tatars was found to be different than those in the Slavic group, though there is a relatively low prevalence of BRCA1 and BRCA2 founder mutations in Slavic populations. This suggests that HBOCS genetic predisposition tests for Tatar patients should be different than those used for Slavic populations. We found a novel c.1047-2A>G nucleotide variant of the CDK12 gene that was strongly associated with HBOCS and present only in HBOCS patients of Tatar ethnic origin. To the best of our knowledge, our study is the first demonstrating that CDK12 c.1047-2A>G nucleotide variation results in HBOCS predisposition, indicating CDK12 involvement in HBOCS.

## Materials and methods

The study cohort comprised of female patients with a familial history of OC and/or BC (HBOCS) as well as healthy donors without a familial history of OC and/or BC obtained from the Republican Clinical Oncology Dispensary of the Ministry of Healthcare of Tatarstan Republic (RCOD MHTR), Volga District of Tatarstan Republic, or the Federal Scientific Clinical Centre of Federal Medical-Biological Agency Russian Federation (FSCC FMBA RF), Moscow, Russia. The clinical and demographic characteristics for the study participants are summarized in Tables 1, 2. The study participants in the Tatar group self-identified as Tatars. The non-Tatar group included participants of unknown or mixed ancestry from Volga District of Tatarstan Republic. The study participants in the Slavic group self-identified with some or several Slavic ethnicities from Moscow, Russian Federation. All participants provided informed consent.

**Table 1:** The demographic characteristics of the participant cohorts.

**Geographic region**	**Healthy donors**		**Sporadic BC and/or OC**		**HBOCS patients**				
	**No**.	**Mean age, years (range)**	**No**.	**Mean age, years (range)**	**All**	**BC**		**OC**	
					**No. (%)**	**No. (%)**	**Mean age, years (range)**	**No. (%)**	**Mean age, years (range)**
Volga District of Tatarstan Republic	238	54 (32–74)	93	56 (32–86)	199 (100)	88 (44)	49 (23–88)	111 (56)	55 (22–86)
Moscow	372	55 (34–78)	80	55 (34–75)	95 (100)	40 (42)	48 (32–72)	45 (58)	52 (30–74)

**Table 2:** The clinical characteristics of the HBOCS patients from Tatarstan Republic.

**Geographic region**	**All patients, No. (%)**	***BRCA1* mutation, No. (%)**	***BRCA2* mutation, No. (%)**	**Mutations in non-*BRCA1/2* genes, No. (%)**	**No mutations or variants of uncertain significance, No. (%)**
Volga District of Tatarstan Republic	199 (100)	54 (27)	24 (12)	22 (11)	99 (50)
	Age at disease manifestation Mean age, years (range)				
	51 (22–88)	48 (28–82)	51 (32–70)	52 (31–79)	54 (22–88)

### DNA isolation

Whole blood samples were collected from all study participants. Genomic DNA was isolated from the blood using the QIAamp DNA Blood Mini QIAcube Kit (Qiagen) and quantified using the NanoVue Plus Spectrophotometer (GE Healthcare).

### Targeted next-generation sequencing (NGS)

Targeted NGS was performed in a cohort of 199 HBOCS patients from the Volga District of the Tatarstan Republic. DNA (100 ng) was used to generate sequencing libraries. The NimbleGen SeqCap EZ Choice kit (“Roche”) was used for target enrichment and sequencing was performed using the Illumina MiSeq (“Illumina”) following the manufacturer's protocol. Raw-data reads were aligned to the human reference genome (hg19) using the aligner BWA (MEM algorithm) with BamQC, FastQC, and NGSrich quality control checks. GATK Haplotype v3.6 was applied for variant calling. Variant Call Format files were annotated using SnpSift & SnpEff, ANNOVAR, and Alamut Batch. MaxEnt, NNSPLICE, and HSF were used as *in silico* splice-prediction tools. The HGMD Professional 2017.1 and BIC databases were used to identify pathogenic nucleotide variants. Prediction of pathogenicity was determined by *in silico* tools SIFT, PolyPhen2, MutationTaster, FATHMM, CADD13, DANN, REVEL. The gene panel included *ATM, BARD1, BRCA1, BRCA2, CDH1, CDK4, CDK12, CDKN2A, CFTR, CHEK1, CHEK2, CTNNA1, EPCAM, FANCI, FANCJ/BRIP1, FANCL, MLH1, MSH2, MSH6, MUTYH, PALB2, PARP1, PDGFRA, PMS2, PPP2R2A, PRSS1, RAD51B, RAD51C, RAD51D, RAD54L, SPINK1, STK11, TP53*, and *XRCC3*.

### RT-PCR assay

RT-PCR analysis was used to assess the presence or absence of a *CDK12* c.1047-2A>G nucleotide variant in 93 patients with sporadic OC and/or BC, 238 healthy participants of Tatar ethnic origin, 95 HBOCS patients, 80 patients with sporadic OC and/or BC, and 372 healthy participants of Slavic ethnic origin. RT-PCR was performed using TaqMan probes (FAM-atttcCtAcTgGaAaa-BHQ-1 for wild-type, VIC-atttcCtAcCgGaAaa-BHQ-2 for c.1047-2A>G mutation) and the following primers: forward 5′-TGGCACTTAATCTATTTTACA-3′, reverse 5′-GGATCTCTTCTTTTTACTATGA-3′. RT-PCR was carried out on a thermal cycler “StepOnePlus” (Applied Biosystems, USA) with a 10 μL final volume containing TurboBuffer (Evrogen, Russia), 400 nM forward and reverse primers, 150 nM probes, 1.5 unit Taq DNA polymerase, and 20–50 ng of genomic DNA. Thermocycling conditions: a first cycle at 95°C for 2 min; 40 cycles at 94°C for 10 s, and 40 cycles at 56°C for 90 s. PCR product size was 200 bp. Analysis of the amplification product was performed with the “end point” detection method using built-in thermocycler software tools accompanying SDS version 1.4. Positive control DNA was used to validate assay sensitivity of and analyzed in parallel with all samples. Presence of *CDK12* c.1047-2A>G nucleotide variation was determined by targeted NGS and confirmed by RT-PCR assay.

### Statistical analysis

Standard statistical tests were used to analyze the data, including a two-tailed Fisher exact test performed with the R software (v.3.3). Statistical significance was defined as a p value less than 0.05. Values was obtained from *fisher.test* function.

## Results

In a group of 199 HBOCS patients from the Volga district, Republic of Tatarstan (106 of Tatar ancestry and 93 of mixed or unknown ancestry) we employed Targeted NGS to detect a total of 38 germline nucleotide variant markers in 8 genes from a panel of 33 genes. The frequencies of the markers are shown in Table 3.

**Table 3:** Germline nucleotide variants in HBOCS patients from Volga District, Republic of Tatarstan and in healthy subjects from Non-Finish European population (NFE).

**Gene**	**Hg19 coordinate**	**Transcript:cDNA**	**Protein**	**N**	**Frequency in HBOCS patients from Tatarstan, %**	**Frequency in NFE**
BRCA1	chr17:41209079	NM_007300.3:c.5329dup (also known as 5382insC)	p.Gln1777Profs^*^74	9	4.5	1.6^*^10^−4^
BRCA1	chr17:41215382	NM_007300.3:c.5224C>T	p.Gln1742^*^	4	2.0	8.9^*^10^−6^
BRCA1	chr17:41258504	NM_007300.3:c.181T>G (also known as T300G)	p.Cys61Gly	2	1.0	6.3^*^10^−5^
BRCA1	chr17:41209095	NM_007300.3:c.5314C>T	p.Arg1772^*^	2	1.0	7.9^*^10^−6^
BRCA2	chr13:32906576	NM_000059.3:c.965_966dup	p.Val323Lysfs^*^2	2	1.0	N/A
CDH1	chr16:68844220	NM_004360.4:c.808T>G	p.Ser270Ala	2	1.0	4.7^*^10^−4^
CHEK2	chr22:29130389	NM_001005735.1:c.319+2T>A	- (splice site)	2	1.0	1.1^*^10^−4^
MUTYH	chr1:45797228	NM_001128425.1:c.1187G>A	p.Gly396Asp	2	1.0	4.8^*^10^−3^
BRCA1	chr17:41246513	NM_007300.3:c.1034_1035insC	p.Pro346Serfs^*^4	1	0.5	0.0
BRCA1	chr17:41245587	NM_007300.3:c.1961del (also known as 2080delA)	p.Lys654Serfs^*^47	1	0.5	6.7^*^10^−5^
BRCA1	chr17:41243924	NM_007300.3:c.3624del	p.Lys1208Asnfs^*^2	1	0.5	N/A
BRCA1	chr17:41245587	NM_007300.3:c.1961del (founder mutation 2080delA)	p.Lys654Serfs^*^47	1	0.5	6.7^*^10^−5^
BRCA1	chr17:41244614	NM_007300.3:c.2934del	p.Arg979Valfs^*^21	1	0.5	N/A
BRCA1	chr17:41244282	NM_007300.3:c.3266del	p.Leu1089Cysfs^*^20	1	0.5	N/A
BRCA1	chr17:41215890	NM_007300.3:c.5215+1G>T	- (splice site)	1	0.5	N/A
BRCA1	chr17:41244761	NM_007300.3:c.2787del	p.Pro930Leufs^*^70	1	0.5	N/A
BRCA1	chr17:41246083	NM_007300.3:c.1465G>T	p.Glu489^*^	1	0.5	N/A
BRCA1	chr17:41245918	NM_007300.3:c.1630del	p.Gln544Lysfs^*^2	1	0.5	N/A
BRCA1	chr17:41246633	NM_007294.3:c.915T>A	p.Cys305^*^	1	0.5	1.8^*^10^−5^
BRCA2	chr13:32900279	NM_000059.3:c.468dup	p.Lys157^*^	1	0.5	6.7^*^10^−5^
BRCA2	chr13:32906625	NM_000059.3:c.1010_1011insTG	p.Asp339Leufs^*^11	1	0.5	N/A
BRCA2	chr13:32907409	NM_000059.3:c.1796_1800del	p.Ser599^*^	1	0.5	9.2^*^10^−6^
BRCA2	chr13:32968950	NM_000059.3:c.9381G>A	p.Trp3127^*^	1	0.5	N/A
BRCA2	chr13:32968836	NM_000059.3:c.9269del	p.Phe3090Serfs^*^14	1	0.5	4.8^*^10^−5^
BRCA2	chr13:32906843	NM_000059.3:c.1231del	p.Ile411Tyrfs^*^19	1	0.5	N/A
BRCA2	chr13:32915113	NM_000059.3:c.6622_6623del	p.Asn2208Tyrfs^*^16	1	0.5	0.0
BRCA2	chr13:32915062	NM_000059.3:c.6574del	p.Met2192Trpfs^*^14	1	0.5	N/A
BRCA2	chr13:32914265	NM_000059.3:c.5773del	p.Gln1925Argfs^*^38	1	0.5	9.0^*^10^−6^
CHEK2	chr22:29091857	NM_001005735.1:c.1229del	p.Thr410Metfs^*^15	1	0.5	2.5^*^10^−6^
CHEK2	chr22:29099504	NM_001005735.1:c.1022_1026del	p.Tyr341Cysfs^*^12	1	0.5	N/A
CHEK2	chr22:29090060	NM_001005735.1:c.1550G>A	p.Arg517His	1	0.5	1.1^*^10^−4^
FANCI	chr15:89838324	NM_001113378.1:c.2635C>T	p.Arg879^*^	1	0.5	1.7^*^10^−5^
MSH2	chr2:47630353	NM_000251.2:c.23C>T	p.Thr8Met	1	0.5	1.6^*^10^−4^
MUTYH	chr1:45800146	NM_001128425.1:c.74G>A	p.Gly25Asp	1	0.5	N/A
MUTYH	chr1:45800167	NM_001128425.1:c.53C>T	p.Pro18Leu	1	0.5	3.1^*^10^−5^
MUTYH	chr1:45798269	NM_001128425.1:c.667A>G	p.Ile223Val	1	0.5	3.4^*^10^−4^
RAD51C	chr17:56801399	NM_058216.2:c.905-2_905-1del	- (splice site)	1	0.5	0.0

* *N/A, not available*.

We also performed Targeted NGS for the *CDK12* gene and identified a c.1047-2A>G nucleotide variant in 8 of the 106 patients of Tatar descent. The presence of c.1047-2A>G in the *CDK12* gene, identified by Targeted NGS, was confirmed by RT-PCR (data not shown). In a cohort of Slavic participants from Moscow, this nucleotide variant was absent in 95 patients with HBOCS, 80 patients with sporadic BC and/or OC, and 372 healthy controls as determined by RT-PCR. In a cohort of participants from the Volga District, Republic of Tatarstan, the frequency of c.1047-2A>G mutation was significantly higher in HBOCS patients compared to healthy controls (9/199 vs. 1/238, *p* = 0.0066, OR = 11.18, CI 95% = 1.53–492.95, Table 4). The cohort of HBOCS patients from the Republic of Tatarstan included 106 patients of Tatar ethnicity, and 93 patients of non-Tatar, mixed, or unknown ethnicity. Given that the Tatars ethnic group is one of the most common in the Republic of Tatarstan, constituting almost 50% of the total population, we assume that about half of the healthy donors randomly recruited to this study in the Tatarstan Republic were also of Tatar ancestry.

**Table 4:** *CDK12* gene c.1047-2A>G nucleotide variant frequency distribution.

**Geographic region**	**Ethnicity**	**HBOCS**		**Sporadic BC and/or OC**	**Healthy controls**	**HBOCS vs. controls (9/199 vs. 1/238)**	**Sporadic BC/OC vs. controls (2/93 vs. 1/238)**
Volga District, Republic of Tatarstan	Tatars	8/106 (7.6%)	9/199 (4.5%)	2/93 (2.2%)	1/238 (0.42%)	*p =* 0.0066 OR = 11.18 CI 95% = 1.53–492.95	*p =* 0.20 OR = 5.07 CI 95% = 0.26–301.34
	Non-Tatars, Mixed or Unknown	1/93 (1.1%)					
Moscow	Slavic	0/95 (0%)		0/80 (0%)	0/372 (0%)	–	–

All HBOCS patients with *in silico* pathogenic mutations of the *CDK12* gene had negative HER2 status.

We also found several other nucleotide variants in the CDK12 gene in the group of HBOCS patients (Table 5), with a deleterious prediction of pathogenicity determined by *in silico* tools (SIFT, PolyPhen2, MutationTaster, CADD, DANN, REVEL). Among the patients with HBOCS harboring CDK12 nucleotide variants determined as pathogenic, 21% also had pathogenic nucleotide variants in BRCA1 gene.

**Table 5:** All *in silico* pathogenic *CDK12* nucleotide variants in HBOCS patients from Volga District, Tatarstan Republic.

**Patient**	**Hg19 coordinate transcript:cDNA protein**	**Frequency in gnomAD NFE(%)**	**Number in our study**	**Frequency in our study(%)**	**Other mutations**	**Immunohistochemistry(%)**			
						**ER**	**PR**	**HER2**	**KI-67**
Pat.1		0.052	9	4.5	BRCA2:NM_000059.3:c.3689C>T:p.Ser1230Phe RAD54L:NM_001142548.1:c.2213G>A:p.Arg738His	8	6	0	20
Pat.2					**BRCA1:NM_007300.3:c.181T**>G:p.Cys61Gly^*^	0	0	0	0
Pat.3					FANCI:NM_001113378.1:c.286G>A:p.Glu96Lys ATM:NM_000051.3:c.5975A>C:p.Lys1992Thr	7	7	0	60
Pat.4	chr17:37627130 NM_016507.3:				BRCA2:NM_000059.3:c.9976A>T:p.Lys3326^*^	-	-	-	-
Pat.5	c.1047-2A>G p.?				Absent	-	-	-	-
Pat.6					Absent	3	4	0	10
Pat.7					**BRCA1:NM_007300.3:c.5224C**>T:p.Gln1742^*^	5	5	0	10
Pat.8					Absent	-	-	-	-
Pat.9					CDKN2A:NM_001195132:c.C496T:p.H166Y MSH6:NM_000179.2:c.2633T>C:p.Val878Ala	?	?	?	?
Pat.10	chr17:37687333 NM_016507.3:c.4237C>T p.His1413Tyr	0.0019	1	0.5	BRCA1:NM_007300.3:c.4946T>C:p.Met1649Thr	8	8	0	0
Pat.11	chr17:37627556 NM_016507.3:c.1471C>T p.Leu491Phe	0.0045	1	0.5	BARD1:NM_000465.3:c.104C>G:p.Ala35Gly	0	0	0	97
Pat.12	chr17:37627187 NM_016507.3:c.1102T>A p.Ser368Thr	0.02	1	0.5	MLH3:NM_001040108.1:c.1870G>C:p.Glu624Gln	7	8	0	30
Pat.13	chr17:37673748 NM_016507.3:c.2902T>C p.Tyr968His	0.0045	1	0.5	BRIP1:NM_032043.2:c.728T>C:p.Ile243Thr	0	0	0	0
Pat.14	chr17:37676286 NM_016507.3:c.3041C>T p.Thr1014Ile	0.0012	1	0.5	**BRCA1:NM_007300.3:c.4327C**>T:p.Arg1443^*^ RAD54L:NM_001142548.1:c.1317G>C:p.Glu439Asp	0	0	0	0

* *“-”- patients with ovarian cancer, receptor status is usually not determined in clinical practice; “?” – patients with breast cancer with unknown receptor status*.

Forty three percent of the patients in HBOCS cohort were HER2 positive, but all patients carrying CDK12 c.1047-2A>G nucleotide variant were HER2 negative (Table 5).

We hypothesized that the c.1047-2A>G nucleotide variant in the *CDK12* gene could potentially affect splicing. *In-silico* splice site prediction analysis of the *CDK12* c.1047-2A>G variant by MaxEnt, NNSPLICE, and HSF tools suggests that the variant is a splice site substitution in the acceptor splice site of intron 1, likely resulting in a skip of exon 2. Therefore, the *CDK12* c.1047-2A>G mutation may lead to production of a shorter alternative splice transcript. Interestingly, we also found several other nucleotide variants in the CDK12 gene in the group of HBOCS patients (Table 5), with a greater than 90% deleterious prediction of pathogenicity determined by *in silico* tools. Among the patients with HBOCS harboring CDK12 nucleotide variants determined as pathogenic, 21% also had pathogenic nucleotide variants in BRCA1 gene.

## Discussion

The Russian population includes many ethnicities, and is characterized by huge genetic diversity. Slavic and non-Slavic ethnicities in Russia may have different profiles of nucleotide variants resulting in HBOCS predisposition. Therefore, it is possible that identification of novel ethno-specific markers will decrease false-negative results of genetic risk assessment. There is a degree of variability in the frequency of HBOCS-associated nucleotide variants in the BRCA1 and BRCA2 genes of non-Caucasian populations (31, 32). Indeed, one of the most common markers in European populations, BRCA1 5382insC, was not found in hereditary BC patients from several non-Slavic indigenous populations (Altaians, Buryats, and Tuvinians) in Russia (31). Our previously published data on germline BRCA1 and BRCA2 nucleotide variants in a small group of Tatar patients with BC indicated the same trend (8). To the best of our knowledge, no data exists on the spectrum of disease-associated nucleotide variants in HBOCS patients of Tatar descent.

We tested multiple-gene panels for the presence of HBOCS predisposition markers in Tatar patients and detected several germline nucleotide variants in the BRCA1, BRCA2, CDK12, CDH1, CHEK2, FANCI, MUTYH, MSH2, and RAD51C genes, including some pathogenic variants previously reported in other populations. Strikingly, their prevalence and spectrum in Tatar HBOCS patients was found to be different to that reported in European populations, particularly in Russia (2, 6, 32).

Currently, nucleotide variants in the *CDK12* gene are not included in panels of HBOCS predisposition markers, despite the fact that several lines of evidence strongly suggest CDK12 involvement in OC and BC pathogenesis. *CDK12* has been found to be one of the most frequently mutated genes in high grade serous OC, harboring mutations in 3% of cases (23). In OC, *CDK12* mutations deregulate expression of HRR pathway genes (33). In BC, *CDK12* is found to be frequently co-amplified with the oncogene ERBB2. Such amplification may contribute to BC pathogenesis (34). Recent breakthroughs in molecular diagnostic techniques have allowed the incorporation of NGS into clinical practice, allowing identification of small deletions/insertions, single nucleotide variants, and other variations in the sequence of candidate genes predisposing patients to various diseases such as HBOCS (34). We proposed that CDK12 is involved in HBOCS and performed a Targeted NGS-based approach to identify disease-associated nucleotide variants of the *CDK12* gene in the Tatar population.

In this study, we detected a novel germline nucleotide variant c.1047-2A>G in the *CDK12* gene in a group of Tatar patients with HBOCS. The percentage of *CDK12* c.1047-2A>G variants in Tatar and non-Tatar patients (106 and 93 patients assessed, respectively) was 4.5%, that is significantly higher than the 0.42% observed in a group of 238 healthy donors of mixed or unknown ancestry (Tatar and non-Tatar) from the same geographical region. One potential weakness of this study is the possibility that the healthy control group consists of primarily non-Tatar participants, which would result in a difference in the c.1047-2A>G nucleotide variant frequency between the HBOCS and control groups solely because the c.1047-2A>G variant occurs more frequently in the Tatar population. However, given that Tatar is one of the major ethnic groups in the Republic of Tatarstan, comprising almost 50% of the total population, we assume that about half of the healthy donors are of Tatar ethnicity. We also recruited a relatively large number of participants in a healthy control group (238 participants), to ensure a cohort that better represents the entire population. The frequency of the *CDK12* c.1047-2A>G nucleotide variant in Tatar patients is relatively high and similar to the frequency of the *BRCA1* 5382insC, a founder-mutation present in many Russian populations. The c.1047-2A>G variant was detected in patients from apparently non-related families. Therefore, it is possible that *CDK12* c.1047-2A>G is a founder mutation in the Tatar population, at least for the Tatar sub-population in the Kazan region. Importantly, carriers of the *CDK12* c.1047-2A>G variant in the group of non-Tatar HBOCS patients from the Volga District were of Chuvash ethnicity, which is closely related to Tatars and belongs to the Turkic ethnic group under which Tatars are classified.

Overall, we conclude that *CKD12* is a candidate gene for HBOCS syndrome. Currently, there is only one other report describing cancer patient carrying the *CDK12* c.1047-2A>G nucleotide variant. Remarkably, it is also a patient with OC found in a cohort of OC patients in USA (35). We propose that *CDK12* is involved in pathogenesis of other malignancies characterized by impaired HRR (10, 12), and that c.1047-2A>G may be associated with such diseases. This indicates that *CDK12* c.1047-2A>G could be used as a diagnostic marker.

Frequencies of this mutation in samples from the Exome Aggregation Consortium database [http://gnomad.broadinstitute.org/](36) are extremely low (Table 6). Nevertheless, it is present in several populations, with highest frequency of 0.1% occurring in South Asian populations. We determined the frequency of *CDK12* c.1047-2A>G mutation in healthy participants from the Volga District of the Republic of Tatarstan to be 0.42%. This raises the question whether c.1047-2A>G should be classified as a mutation or a nucleotide polymorphism (37). Therefore, we define c.1047-2A>G as a nucleotide variant and classify it as pathogenic in accordance with recommendations of the American College of Medical Genetics and Genomics (ACMG) (38).

**Table 6:** *CDK12* gene c.1047-2A>G nucleotide variant frequencies in populations (Genome Aggregation Database).

**Population**	**Allele number**	**Allele frequency**
South Asian	26564	0.1%
European (Non-Finnish)	120204	0.05%
European (Finnish)	25106	0.02%
African	23590	0.004%
Latino	28642	0.003%
Ashkenazi Jewish	8670	0%
East Asian	17724	0%

The Tatar population in the Volga region has low interpopulation differentiation (39), which indicates that the results of the current study may be extrapolated to the whole Tatar population in the Volga region of the Republic of Tatarstan. Importantly, Tatars who live in the eastern regions of Tatarstan have genetic similarity to the Bashkirs ethnic group (39). Thus, we expect that the c.1047-2A>G nucleotide variant in the *CDK12* gene might be involved in HBOCS in individual of Bashkirs ethnicity as well, which should be addressed in further studies. The relatively high percentage of c.1047-2A>G among healthy participants in our study may have several explanations. There is a possibility that even if asymptomatic carriers of c.1047-2A>G have not developed the disease yet, they eventually will. Alternatively, c.1047-2A>G may result in a “disease predisposing” phenotype, but the second mutation, present among patients but is absent in healthy controls, is necessary to trigger the disease as delineated by the “two hit” hypothesis (40). Finally, carriers of the c.1047-2A>G nucleotide variant in healthy group may also harbor “protective” nucleotide variant(s) (yet unknown), which neutralize the pathogenic effect of c.1047-2A>G (41). Identifying such protective nucleotide variants would open an avenue for new therapeutic strategies.

The *CDK12* gene is located on chromosome 17q12 and is comprised of 14 exons. Currently, there are two identified isoforms of the *CDK12* gene, a shorter and longer isoform, differing in one exon. The shorter splice isoform results in an 1481 amino acid protein and the longer splice isoform encodes an 1,490 amino acid protein, with both harboring the same functional domains (22). It should be noted that mutations introducing a new splice-site sequence may result in loss of functional domains or altered folding of the CDK12 protein. The c.1047-2A>G mutation in the *CDK12* gene may alter splicing. We speculate that the c.1047-2A>G variant results in a truncated CDK12 protein and loss of function, leading to impaired HRR. It has previously been shown that CDK12 protein inactivation results in cells more sensitive to genotoxic insult and that tumors with an HRR pathway deficiency are highly sensitive to poly(ADP-ribose) polymerase (PARP) inhibitors. In particular, inactivation of CDK12 in OC cells sensitizes them to the DNA cross-linking agent cisplatin and to PARP inhibitors such as veliparib and olaparib (42, 43). In BC, pharmacological inhibition of CDK12 reverses PARP inhibitor resistance in both BRCA wild-type and BRCA-mutant cells (44). If carriers of the c.1047-2A>G nucleotide variant have non-functional CDK12 protein, they may exhibit increased sensitivity to PARP inhibitors.

*CDK12* gene is located in close proximity to the oncogene *ERBB2*, also known as *HER2*. In BC, *CDK12* is frequently co-amplified with the *HER2* (34). Previously, a correlation of HER2 status and CDK12 level was found in a cohort of BC patients. In most of the HER2 amplified tumors level of CDK12, both mRNA and protein, was high, while absence of CDK12 was rarely observed (45). While 43% of patients in the cohort were HER2 positive, all patients harboring pathogenic nucleotide variants in *CDK12* were HER2 negative. Whether HER2 negative status is a functional consequence of the presence of pathogenic nucleotide variants in *CDK12* is beyond the scope of current research, but should be addressed in future studies.

Overall, our study demonstrates that prevalence of disease-associated mutations in the *BRCA1* and *BRCA2* genes in the Russian population is significantly different in patients of Tatar and Slavic ethnic origins. We identified the c.1047-2A>G germline nucleotide variant in the *CDK12* gene, which may result in an alternative *CDK12* splice variant and is strongly associated with HBOCS. We recommend that this variant become part of the standard testing panel for HBOCS susceptibility markers in Tatar patients with a family history of OC and BC. Incorporation of the c.1047-2A>G marker in this genetic diagnostic panel may also lead to improved therapeutic strategies, such as stratification of the patients according to potential sensitivity to PARP inhibitors. This finding also confirms the role of *CKD12* as a candidate gene for HBOCS predisposition.

## Ethics statement

This study was carried out in accordance with the recommendations of International code of medical ethics, ethical committee of Federal Research and Clinical Center of Federal Meadical and Biological Agency of Russia. The protocol was approved by the ethical committee of Federal Research and Clinical Center of Federal Meadical and Biological Agency of Russia. All subjects gave written informed consent in accordance with the Declaration of Helsinki.

## Author contributions

AN, DK, ES, LS, OB, OG, MD, MG, and RE data collection, analysis and interpretation. AN, DC, ES, OB, and OG study conception and design. AN and DC drafting and critical revision of the manuscript.

### Conflict of interest statement

The authors declare that the research was conducted in the absence of any commercial or financial relationships that could be construed as a potential conflict of interest.

## References

[1] Torre LA Bray F Siegel RL Ferlay J Lortet-Tieulent J Jemal A. Global cancer statistics, 2012. CA Cancer J Clin. (2015) 65:87–108. 10.3322/caac.21262 25651787

[2] Sokolenko AP Iyevleva AG Mitiushkina NV Suspitsin EN Preobrazhenskaya EV Kuligina ES. Hereditary breast-ovarian cancer syndrome in Russia. Acta Naturae (2010) 2:31–5. 22649661 PMC3347586

[3] Cho S-H Jeon J Kim SI. Personalized medicine in breast cancer: a systematic review. J Breast Cancer (2012) 15:265–72. 10.4048/jbc.2012.15.3.265 23091538 PMC3468779

[4] Jing L Su L Ring BZ. Ethnic background and genetic variation in the evaluation of cancer risk: a systematic review. PloS ONE (2014) 9:e97522. 10.1371/journal.pone.0097522 24901479 PMC4046957

[5] Gayther SA Harrington P Russell P Kharkevich G Garkavtseva RF Ponder BA. Frequently occurring germ-line mutations of the BRCA1 gene in ovarian cancer families from Russia. Am J Hum Genet. (1997) 60:1239–42. 9150173 PMC1712436

[6] Sokolenko AP Rozanov ME Mitiushkina NV Sherina NY Iyevleva AG Chekmariova EV. Founder mutations in early-onset, familial and bilateral breast cancer patients from Russia. Fam Cancer (2007) 6:281–6. 10.1007/s10689-007-9120-5 17333477

[7] Tereschenko IV Basham VM Ponder BAJ Pharoah PDP. BRCA1 and BRCA2 mutations in Russian familial breast cancer. Hum Mutat. (2002) 19:184. 10.1002/humu.9008 11793480

[8] Shagimardanova E Shigapova L Gusev O Nikitin A Druzhkov M Enikeev R Germline BRCA screening in breast cancer patients in Tatar women population. Ann Oncol. (2016) 27:vi43–67. 10.1093/annonc/mdw364.26

[9] Negrini S Gorgoulis VG Halazonetis TD. Genomic instability - an evolving hallmark of cancer. Nat Rev Mol Cell Biol. (2010) 11:220–8. 10.1038/nrm2858 20177397

[10] Bristow RG Ozcelik H Jalali F Chan N Vesprini D. Homologous recombination and prostate cancer: a model for novel DNA repair targets and therapies. Radiother Oncol J Eur Soc Ther Radiol Oncol. (2007) 83:220–30. 10.1016/j.radonc.2007.04.016 17531338

[11] Konstantinopoulos PA Ceccaldi R Shapiro GI D'Andrea AD. Homologous recombination deficiency: exploiting the fundamental vulnerability of ovarian cancer. Cancer Discov. (2015) 5:1137–54. 10.1158/2159-8290.CD-15-0714 26463832 PMC4631624

[12] Nilles N Fahrenkrog B. Taking a bad turn: compromised DNA damage response in Leukemia. Cells (2017) 6:11. 10.3390/cells6020011 28471392 PMC5492015

[13] Kitagishi Y Kobayashi M Matsuda S. Defective DNA repair systems and the development of breast and prostate cancer (review). Int J Oncol. (2013) 42:29–34. 10.3892/ijo.2012.1696 23151935

[14] Hoeijmakers JHJ. Genome maintenance mechanisms for preventing cancer. Nature (2001) 411:366–74. 10.1038/35077232 11357144

[15] Davis AJ Chen DJ. DNA double strand break repair via non-homologous end-joining. Transl Cancer Res. (2013) 2:130–43. 10.3978/j.issn.2218-676X.2013.04.02 24000320 PMC3758668

[16] Meindl A Hellebrand H Wiek C Erven V Wappenschmidt B Niederacher D. Germline mutations in breast and ovarian cancer pedigrees establish RAD51C as a human cancer susceptibility gene. Nat Genet. (2010) 42:410–4. 10.1038/ng.569 20400964

[17] Li J Meeks H Feng B-J Healey S Thorne H Makunin I. Targeted massively parallel sequencing of a panel of putative breast cancer susceptibility genes in a large cohort of multiple-case breast and ovarian cancer families. J Med Genet. (2016) 53:34–42. 10.1136/jmedgenet-2015-103452 26534844 PMC4915734

[18] Easton DF Pharoah PDP Antoniou AC Tischkowitz M Tavtigian SV Nathanson KL. Gene-Panel Sequencing and the Prediction of Breast-Cancer Risk. N Engl J Med. (2015) 372:2243–57. 10.1056/NEJMsr1501341 26014596 PMC4610139

[19] Mantere T Tervasmäki A Nurmi A Rapakko K Kauppila S Tang J. Case-control analysis of truncating mutations in DNA damage response genes connects TEX15 and FANCD2 with hereditary breast cancer susceptibility. Sci Rep. (2017) 7:681. 10.1038/s41598-017-00766-9 28386063 PMC5429682

[20] Nielsen FC van Overeem Hansen T Sørensen CS. Hereditary breast and ovarian cancer: new genes in confined pathways. Nat Rev Cancer (2016) 16:599–612. 10.1038/nrc.2016.72 27515922

[21] Hollestelle A Wasielewski M Martens JWM Schutte M. Discovering moderate-risk breast cancer susceptibility genes. Curr Opin Genet Dev. (2010) 20:268–76. 10.1016/j.gde.2010.02.009 20346647

[22] Chilà R Guffanti F Damia G. Role and therapeutic potential of CDK12 in human cancers. Cancer Treat Rev. (2016) 50:83–8. 10.1016/j.ctrv.2016.09.003 27662623

[23] Cancer Genome Atlas Research Network Integrated genomic analyses of ovarian carcinoma. Nature (2011) 474:609–15. 10.1038/nature10166 21720365 PMC3163504

[24] Popova T Manié E Boeva V Battistella A Goundiam O Smith NK. Ovarian cancers harboring inactivating mutations in CDK12 display a distinct genomic instability pattern characterized by large tandem duplications. Cancer Res. (2016) 76:1882–91. 10.1158/0008-5472.CAN-15-2128 26787835

[25] Mertins P Mani DR Ruggles KV Gillette MA Clauser KR Wang P. Proteogenomics connects somatic mutations to signalling in breast cancer. Nature (2016) 534:55–62. 10.1038/nature18003 27251275 PMC5102256

[26] Dai Q Lei T Zhao C Zhong J Tang Y Chen B. Cyclin K-containing kinase complexes maintain self-renewal in murine embryonic stem cells. J Biol Chem. (2012) 287:25344–52. 10.1074/jbc.M111.321760 22547058 PMC3408147

[27] Li X Chatterjee N Spirohn K Boutros M Bohmann D. Cdk12 is a gene-selective RNA polymerase II kinase that regulates a subset of the transcriptome, including Nrf2 Target Genes. Sci Rep. (2016) 6:21455. 10.1038/srep21455 26911346 PMC4766476

[28] Cheng SWG Kuzyk MA Moradian A Ichu TA Chang VCD Tien JF. Interaction of cyclin-dependent kinase 12/CrkRS with cyclin K1 is required for the phosphorylation of the C-terminal domain of RNA polymerase II. Mol Cell Biol. (2012) 32:4691–704. 10.1128/MCB.06267-11 22988298 PMC3486194

[29] Blazek D Kohoutek J Bartholomeeusen K Johansen E Hulinkova P Luo Z. The Cyclin K/Cdk12 complex maintains genomic stability via regulation of expression of DNA damage response genes. Genes Dev. (2011) 25:2158–72. 10.1101/gad.16962311 22012619 PMC3205586

[30] Blazek D. The cyclin K/Cdk12 complex: an emerging new player in the maintenance of genome stability. Cell Cycle Georget Tex. (2012) 11:1049–50. 10.4161/cc.11.6.19678 22391210 PMC3335913

[31] Cherdyntseva NV Pisareva LF Ivanova AA Panferova YV Malinovskaya EA Odintsova IN. [Ethnic aspects of hereditary breast cancer in the region of Siberia]. Vestn Ross Akad Med Nauk (2014) 72–79. 25971130 10.15690/vramn.v69i11-12.1186

[32] Sokolenko AP Mitiushkina NV Buslov KG Bit-Sava EM Iyevleva AG Chekmariova EV. High frequency of BRCA1 5382insC mutation in Russian breast cancer patients. Eur J Cancer Oxf Engl. (2006) 42:1380–4. 10.1016/j.ejca.2006.01.050 16737811

[33] Ekumi KM Paculova H Lenasi T Pospichalova V Bösken CA Rybarikova J. Ovarian carcinoma CDK12 mutations misregulate expression of DNA repair genes via deficient formation and function of the Cdk12/CycK complex. Nucleic Acids Res. (2015) 43:2575–89. 10.1093/nar/gkv101 25712099 PMC4357706

[34] Tien JF Mazloomian A Cheng S-WG Hughes CS Chow CCT Canapi LT. CDK12 regulates alternative last exon mRNA splicing and promotes breast cancer cell invasion. Nucleic Acids Res. (2017) 45:6698–716. 10.1093/nar/gkx187 28334900 PMC5499812

[35] Swisher E Evaluation of DNA Repair Function as a Predictor of Response in a Clinical Trial of PARP Inhibitor Monotherapy for Recurrent Ovarian Carcinoma. Seattle, WA: University of Washington (2015).

[36] Lek M Karczewski KJ Minikel EV Samocha KE Banks E Fennell T. Analysis of protein-coding genetic variation in 60,706 humans. Nature (2016) 536:285–91. 10.1038/nature19057 27535533 PMC5018207

[37] Karki R Pandya D Elston RC Ferlini C. Defining “mutation” and “polymorphism” in the era of personal genomics. BMC Med Genomics (2015) 8:37. 10.1186/s12920-015-0115-z 26173390 PMC4502642

[38] Richards S Aziz N Bale S Bick D Das S Gastier-Foster J. Standards and guidelines for the interpretation of sequence variants: a joint consensus recommendation of the American College of Medical Genetics and Genomics and the Association for Molecular Pathology. Genet Med Off J Am Coll Med Genet (2015) 17:405–24. 10.1038/gim.2015.30 25741868 PMC4544753

[39] Denisova GA Malyarchuk BA Derenko MV Kravtsova OA. Population structure of Volga Tatars inferred from the mitochondrial DNA diversity data. Russ J Genet. (2011) 47:340–6. 10.1134/S1022795411020086 21542308

[40] Knudson AG. Two genetic hits (more or less) to cancer. Nat Rev Cancer (2001) 1:157–62. 10.1038/35101031 11905807

[41] Pérez-Gracia JL Ruiz-Ilundáin MG Cancer protective mutations: looking for the needle in the haystack. Clin Transl Oncol. (2001) 169–71.

[42] Bajrami I Frankum JR Konde A Miller RE Rehman FL Brough R. Genome-wide profiling of genetic synthetic lethality identifies CDK12 as a novel determinant of PARP1/2 inhibitor sensitivity. Cancer Res. (2014) 74:287–97. 10.1158/0008-5472.CAN-13-2541 24240700 PMC4886090

[43] Joshi PM Sutor SL Huntoon CJ Karnitz LM. Ovarian cancer-associated mutations disable catalytic activity of CDK12, a kinase that promotes homologous recombination repair and resistance to cisplatin and poly(ADP-ribose) polymerase inhibitors. J Biol Chem. (2014) 289:9247–53. 10.1074/jbc.M114.551143 24554720 PMC3979363

[44] Johnson SF Cruz C Greifenberg AK Dust S Stover DG Chi D. CDK12 Inhibition Reverses De Novo and Acquired PARP Inhibitor Resistance in BRCA Wild-Type and Mutated Models of Triple-Negative Breast Cancer. Cell Rep (2016) 17:2367–81. 10.1016/j.celrep.2016.10.077 27880910 PMC5176643

[45] Naidoo K Wai PT Maguire SL Daley F Haider S Kriplani D. Evaluation of CDK12 Protein Expression as a Potential Novel Biomarker for DNA Damage Response-Targeted Therapies in Breast Cancer. Mol Cancer Ther. (2018) 17:306–15. 10.1158/1535-7163.MCT-17-0760 29133620 PMC6284786

